# Influence of preventive remineralizing techniques on surface roughness and volume loss of dentin submitted to erosive and/or abrasive challenges

**DOI:** 10.4317/jced.61006

**Published:** 2024-03-01

**Authors:** Carla-Silva Carvalho, Isabela-Ribeiro Madalena, Erika-Calvano Kuchler, Maria-Angélica-Hueb de Menezes-Oliveira, Vinícius-Rangel-Geraldo Martins, Denise-Tornavoi de Castro, Juliana-Jendiroba Faraoni, Regina-Guenka Palma-Dibb, Cesar-Penazzo Lepri

**Affiliations:** 1 Department of Biomaterials, University of Uberaba, Uberaba, MG, Brazil; 2 School of Dentistry, Presidente Tancredo de Almeida Neves University Center, São João del Rei, MG, Brazil; 3 Department of Restorative Dentistry, School of Dentistry of Ribeirão Preto, University of São Paulo, Ribeirão Preto, SP, Brazil

## Abstract

**Background:**

The objective this study was to evaluate the influence of preventive remineralizing techniques on surface roughness and volume loss of dentin submitted to erosive and/or abrasive challenges.

**Material and Methods:**

One hundred and eighty specimens of bovine root dentin were made; half of each was isolated (without treatment - WT) and half was subjected to the following remineralizing techniques: fluoride varnish (FV); Regenerate Boosting Serum® (RBS); Er,Cr:YSGG laser (L); fluoride varnish+laser (FV+L); Regenerate Boosting Serum®+laser (RBS+L). The specimens were submitted to erosive, abrasive and erosive followed by abrasive challenge. Erosion was carried out for 5 minutes, twice a day for 10 days. Abrasion was performed with an electric toothbrush and slurry solution for 60 seconds. The evaluation was performed by confocal laser scanning microscopy. Analysis of variance and Tukey tests were used for surface roughness; volume loss comparison was performed using the Kruskal-Wallis test and Dunn’s post-hoc (*p*<0.05).

**Results:**

There was no statistically significant difference in the surface roughness of the reference area in relation to the areas submitted to different types of treatment and challenges (*p*>0.05). Regarding volume loss, the untreated group submitted to erosive/abrasive challenges showed greater percentage of volume loss compared to the other groups (*p*<0.05).

**Conclusions:**

It is concluded that preventive remineralizing techniques are effective in maintaining dentin volume after erosive/abrasive challenges.

** Key words:**YSGG lasers, Dentin, Erosion, Tooth Abrasion.

## Introduction

Dental erosion is a multifactorial condition characterized by loss of tooth structure due to oral acidity unrelated to the accumulation of dental biofilm (1). It depends on the acid source to be divided into extrinsic erosion (generally caused by dietary acids) and intrinsic erosion (caused by endogenous acid) (2). The presence and complexity of erosion defects can still be influenced by several chemical, biological and behavioral parameters (2). A significant increase in the prevalence of dental erosion has been noted over the years (3). Consequences related to dental erosion can be cited, such as dentin hypersensitivity, mechanical wear, occlusal changes, exposure of the dental pulp and aesthetic-functional impairment (1,4).

The surface that has undergone some process of dental erosion becomes more susceptible to dental abrasion (5). Dental abrasion can be characterized by the loss of dental tissue due to the friction of abrasive external objects and/or compounds against tooth surfaces (1). Although toothbrushes, toothpastes and brushing techniques are the main players in the etiology of abrasion, it is difficult to reach a definitive conclusion, as other factors can also contribute to the development of injuries (6). In this context, substantial evidence has revealed new insights to diagnose the early stages of non-carious lesions and allow new preventive approaches to control their progression (3,7-9).

Stern *et al*. (10) demonstrated for the first time that hard tissues could increase their resistance with the use of laser. Recent evidence demonstrates its effectiveness in preventing demineralization of dental structures, especially when subablative parameters are applied, leading to chemical and physical changes (11-13). The Er,Cr:YSGG (erbium, chromium: yttrium, scandium, gallium, garnet) laser has a wavelength of 2.78 µm that is well absorbed by water and by the hydroxyl ions of hydroxyapatite (11). This laser is recommended for cavity preparations, carious tissue removal, soft tissue surgeries, among other applications (8,14-16). The use of lasers to increase dentin acid resistance is promising (7,8,13,17). However, established parameters that can promote satisfactory results are still needed. Thus, the objective of the present work is to evaluate how preventive remineralizing techniques influence the surface roughness and the volume loss of dentin submitted to the erosive and/or abrasive challenges.

## Material and Methods

-Ethical Aspects

This study was conducted and reported according to Declaration of Helsinki and was approved by the Ethical Committee of the University of Uberaba (#003/2021).

-Experimental Design

**Figure 1:**
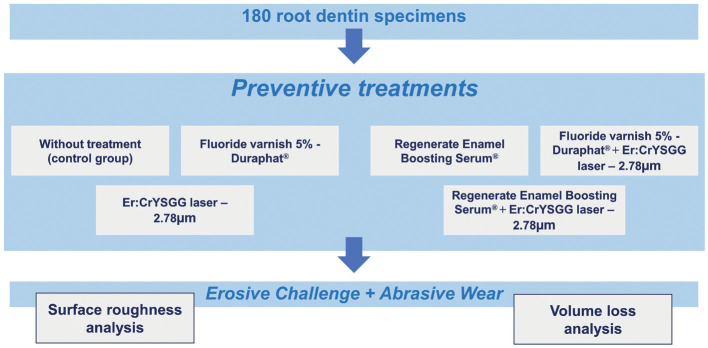
Illustrative flowchart demonstrating the random division of specimens according to the treatments performed.

The experimental units of the study were 90 bovines root dentin without cracks or wear. They were divided in half, origination 180 specimens that were prepared and randomly allocated into 6 different groups, according to the preventive treatments (Table 1). The sample size was calculated considering a significance level of 5% and test power of 85%. Then, the specimens were randomly subdivided and submitted to demineralizing challenges: erosion, performed with soft drink; abrasion, performed with fluoride toothpaste; and erosion followed by abrasion challenges (Fig. 1). Quantitative variables were analysis of surface roughness (SR - μm2) and evaluation of volume loss (VL - μm3).

**Table 1:**
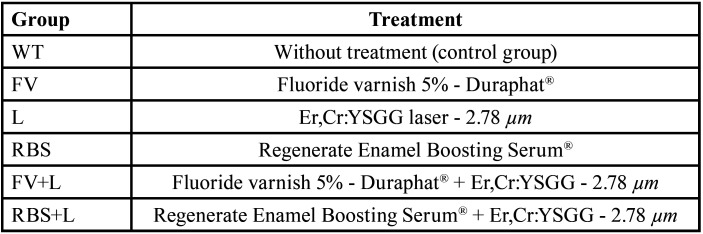
Experimental design.

-Preparation of specimens

The teeth were cleaned and immersed in a 10% formaldehyde solution for disinfection (pH = 7) for 7 days. Then, the teeth were washed and stored in distilled and deionized water at a temperature of 4ºC, which was daily changed for a period of 14 days (7,8).

The coronal portion of the root was sectioned with a diamond disc under refrigeration in an ISOMET 1000® cutting machine (Precision Saw Buehler, Lake Bluff, USA). The first cut was made 1 mm below the cementoenamel junction; the second cut was made in the buccolingual direction, resulting in two halves, each half was again sectioned to obtain specimens with dimensions of 4.5 mm x 4.5 mm x 2.5 mm. The faces of the specimens were sanded with an Arotec APL-4 polisher (Series 41042, Arotec SA Indústria e Comércio, Cotia, São Paulo, Brazil) with #600 sandpaper under water cooling, resulting in a surface area of 18 mm2. The outer (buccal) surface of the specimen was not polished. A positive or negative variation of 10% in the dimensions was allowed. Half of the surface of each specimen was covered with electrical tape. Two layers of red nail polish (Risqué Maybelline Ltda, São Paulo, São Paulo, Brazil) and sculpting wax (Kota Industria, Cotia, São Paulo, Brazil) were applied, isolating the area. After this procedure, the adhesive tape was removed and each specimen had a control surface coated with nail polish and wax protection. The specimens were stored in distilled water at a temperature of 4ºC until the preventive treaments be performed (7,8).

-Specimen treatment

The fluoride varnish 5% (Duraphat®, Colgate Palmolive Ltda, São Paulo, São Paulo, Brazil) was actively applied to the dentin surface with a microbrush (KG Brush, KG Sorensen, Cotia, Sao Paulo, Brazil) for 4 minutes. After this time, the excess was removed with sterile gauze. Regenerate Enamel Boosting Serum® (Unilever, Le Meux, France) was applied with a microbrush (KG Brush, KG Sorensen, Cotia, Sao Paulo, Brazil) for 3 minutes and excess was removed with sterile gauze.

-Laser parameters

Er,Cr:YSGG laser (Biolase Tecnology Inc., San Clemente, USA) was irradiated with a wavelength of 2780 nm, a frequency of 5.0 Hz and a fiber having a diameter of 600 μm (Waterlase laser tip, Biolase ZipTip MZ6 3mm, California, USA). Irradiation was performed at 0.5 W for 10 seconds at 1 mm distance from the target tissue. Water cooling was not used. Then, the specimens were stored in distilled water at 37°C.

-Erosive Challenge 

The erosive challenge was performed with soft drink (Coca-Cola®, Uberlandia Refrescos LTDA, Uberlandia, MG, Brazil), pH = 2.43 at 4oC. Each group was immersed in 50 mL of soft drink for 5 minutes under magnetic stirring. The procedure was repeated twice a day, with an interval of 2 hours, for 10 days. The erosive solution was discarded at each new cycle and the specimens were kept in distilled water at 37ºC between cycles. At the end of the erosive challenge, the specimens were washed with distilled water for 10 seconds (8).

-Abrasive Wear

The abrasive challenge was performed with fluoride toothpaste (Colgate Total 12 Whitening®, Colgate-Palmolive Company, New York, NY, USA) diluted in distilled water in a 1:2 ratio (slurry) and electric toothbrushes, soft bristles and rounded ends (Oral -B Professional Care 5000, Procter and Gamble, Marktheidenfeld, Germany) simulating the oscillatory brushing technique. The brush head has three sets of soft bristles with different shapes and were positioned at different angles and heights. The bristles touched the dentin surface with a force of 2.0 N (≈200g) for 1 minute (166 oscillations/s); every 10 seconds the slurry solution was injected between the bristles. The electric toothbrush was attached to a device during the abrasive challenge. After each brushing, the specimens were washed with distilled water for 10 seconds, slightly dried with absorbent paper, kept in distilled water and stored at 37ºC in the oven. For specimens from groups E+A, brushing occurred 1 hour after the second daily erosive cycle (8).

-Surface Roughness and Volume Loss Analyzes

The specimens were analyzed using a laser scanning confocal microscope. The nail polish and wax from each specimen were removed using a Lecron spatula (Duflex Instruments, Juiz de Fora, Minas Gerais, Brazil). The specimens were placed in distilled water, inserted into an ultrasonic device (Ultrasonic Cleaner 740D - Odontobrás - Ribeirão Preto, São Paulo, Brazil), agitaded for 5 minutes and positioned parallel to the laser scanning microscope Table. After selecting the central region of the specimen, images were acquired using an objective lens of 20x magnification, generating a final magnification of 432x. Through the images, surface roughness and volume loss (%) were analyzed. Data were obtained in μm2 (surface roughness) and μm3 (volume loss) and to carry out statistical calculations, it was transformed into a percentage of lost volume (8).

-Statistical Analysis

The Statistical Software used was SPPS, version 17.1. Data followed a normal distribution (Kolmogorov-Smirnov test) and homogeneous distribution (Levene test). The mean surface roughness values of the different groups were compared using the ANOVA parametric statistical test and Tukey post-hoc. The percentage values of volume loss (%) were submitted to the Kruskal-Wallis non-parametric statistical test and Dunn’s post-hoc. The significance level adopted was 5% (α = 0.05).

## Results

The surface roughness results are described in Table 2. There was no statistically significant difference in the surface roughness of the reference area in relation to the areas submitted to different types of treatment and challenges (*p*>0.05).

**Table 2:**
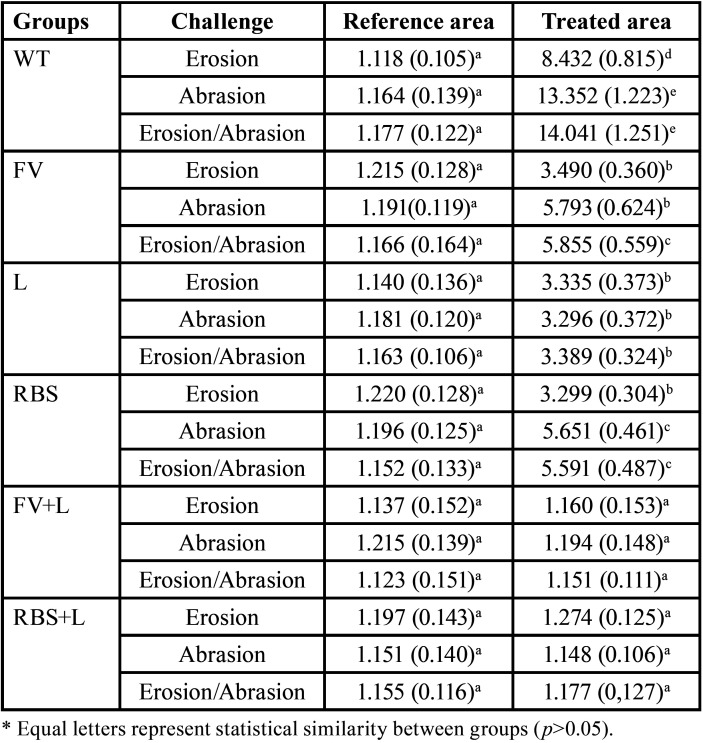
Surface roughness (µm2) mean values (standard deviation) of the groups, considering the reference area and the pre-treated area followed by the demineralization/remineralization cycles.

Regarding volume loss, the WT group submitted to erosive and abrasive challenges showed a statistically significant difference from the other groups (*p*<0.05) Table 3.

**Table 3:**
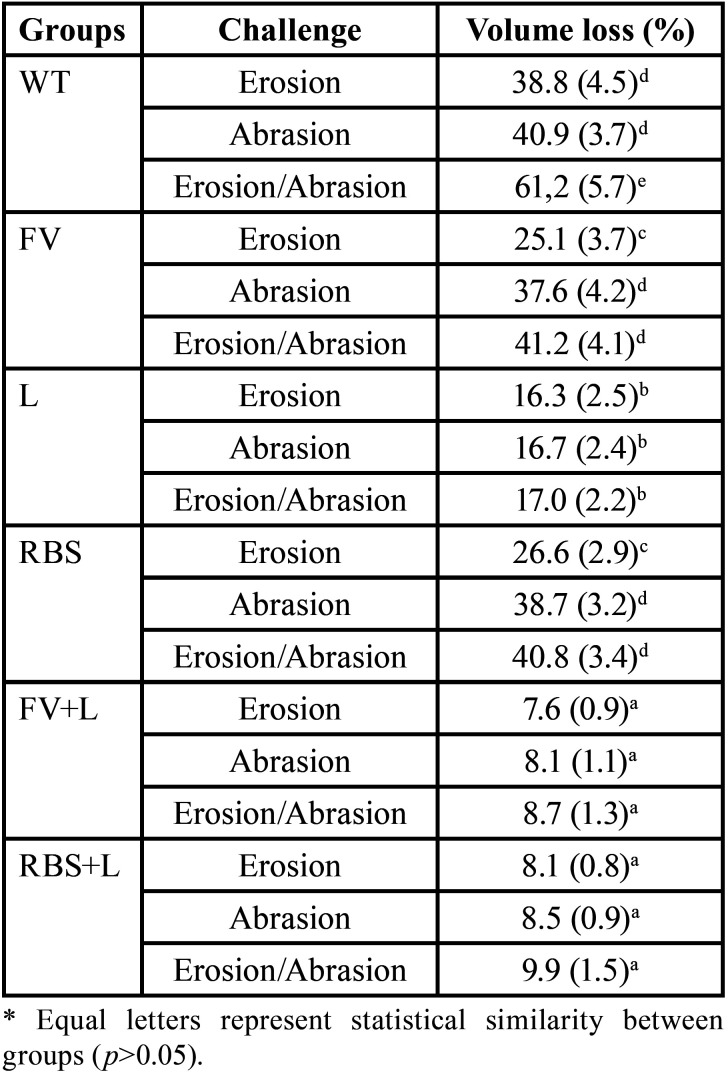
Volume loss mean values (standard deviation) of the groups.

## Discussion

Scientific evidence suggests promising results regarding the use of remineralizing techniques associated with the Er;Cr:YSGG laser, considering that dentin acid resistance was increased (3,7-9,11-13,17). Thus, the present study aimed to evaluate preventive remineralizing techniques associated or not with the Er;Cr:YSGG laser in the surface roughness and volume loss of dentin submitted to erosive and/or abrasive challenges. Our results demonstrate a statistically significant difference as regards volume loss. Therefore, the null hypothesis was rejected.

Remineralizing techniques and its association with Er,Cr:YSGG laser can reduce mineral loss through chemical and morphological changes promoted on the surface of dental tissues subjected to erosion/abrasion (3,4,7-9,11-13,17). It is estimated that preventive treatment can create a protective barrier, which is first removed by dental erosion and, in a second moment, only the effects of abrasive wear occur. That is, the effects of potential erosion/abrasion are minimized. Such suggestions were evidenced in the present work; the untreated group subjected to erosion/abrasion showed a greater loss of surface volume than the other groups that received some type of treatment or were individually submitted to erosive and abrasive challenges.

Regarding the use of Er,Cr:YSGG laser, several studies corroborate with these findings and also highlight a reduced loss of dentin structure when the surface was submitted to erosive and/or abrasive challenges (7,8,11-13,17) .In the present research, the Er,Cr:YSGG laser was used because it has a wavelength equal to 2.78 µm and is well absorbed by water and hydroxyl ions of hydroxyapatite, components present in dentin (11). Furthermore, it is indispensable to use sub-ablative parameters because this type of laser acts through explosive thermo-mechanical ablation (18). Considering that the purpose of the presente study was to prevent volume loss, the ablation would be a undesired effect. When sub-ablative parameters are applied, laser light is absorbed and converted into heat, decreasing tissue solubility. This consequently increase the dentin acid resistance (12,19).

In relation to surface roughness, although the statistical difference could not be observed, the values of surface roughness in the groups that had as preventive treatment the use of fluoride varnish and Regenerate Enamel Boosting Serum® associated with the Er,Cr:YSGG laser (FV+L and RBS+L) showed a lower surface roughness in the different challenges, demonstrating that when the surface is not treated, the erosive and/or abrasive challenges promote rougher areas compared to those that received preventive treatments. Accordingly, our results corroborate with Ana *et al*. (20) and Chiga *et al*. (21). It is noteworthy to developed new studies to minimize rough surfaces of the oral cavity, leading to the reduction of dental biofilm (22,23). Dental biofilm can contribute to the development of other oral health problems such as dental caries and periodontal disease (24).

The surface roughness and dentine volume loss were evaluated using confocal laser scanning microscopy. The use of this technology allows better observation of the morphological characteristics of dentin through high-resolution images (8), enabling analysis of dentinal tubules and areas of demineralization. (7) The profilometric analysis is more sensitive to expose changes resulting from erosive and abrasive challenges, in addition, it does not produce grooves because there is no preparation and no contact with the specimens (25), thus becoming the best choice for evaluating in this study.

## Conclusions

Preventive remineralizing techniques are effective in maintaining dentin volume after erosive/abrasive challenges.
